# Prospective Clinical Study with New Materials for Tissue Regeneration: A Study in Humans

**DOI:** 10.1055/s-0042-1753453

**Published:** 2022-10-04

**Authors:** Nathalie Jeannette Kollek, Carlos Pérez-Albacete Martínez, José Manuel Granero Marín, José Eduardo Maté Sánchez de Val

**Affiliations:** 1 Department of Implant Dentistry, International Dental Research Institute, Catholic University San Antonio of Murcia (UCAM), Murcia, Spain; 2 Department of Integrated Clinic for Adults, International Dental Research Institute, Catholic University San Antonio of Murcia, Murcia, Spain; 3 Department of Restorative Dentistry, International Dental Research Institute, Catholic University San Antonio of Murcia (UCAM), Murcia, Spain; 4 Department of Implant Dentistry and Periodontology, International Dental Research Institute, Catholic University San Antonio of Murcia, Murcia, Spain; 5 Department of Materials Science and Engineering, International Research Institute for Biomaterials, Catholic University San Antonio of Murcia, Murcia, Spain

**Keywords:** bone graft substitute, calcium phosphate, biomaterials, PLGA membrane, collagen membrane, osteointegration

## Abstract

**Objective** This study was performed to evaluate the clinical, radiographic, and histomorphometric outcomes of novel bone grafting materials and dental membranes and to compare the results with current data from the literature.

**Materials and Methods** New synthetic bone substitutes, consisting of biphasic calcium phosphate in the ratio of 60% hydroxyapatite and 40% β-tricalcium phosphate, were applied in bony defects and covered by either a novel synthetic poly(lactic-co-glycolic) acid (PLGA) or porcine collagen membrane. A sample of 51 biomaterials was placed in a total of 20 patients during different surgical protocols. Implants were simultaneously inserted, and in the case of sinus floor elevations 6 months later. Pre- and postoperative cone-beam computed tomographies were taken. Bone biopsies were harvested from augmented sides and processed for histomorphometric evaluation.

**Statistical Analysis** Averages and ranges were calculated for the percentage of newly formed bone, residual biomaterial, and connective tissue. Data were submitted to analyze the radiological mean differences in length, width, and density. Paired *t* -tests were deployed for the analysis of differences within each group between the baseline (preoperative) and the final (postoperative) measurements.

**Results** The mean bone gain in length and width were 0.96 ± 3.33 mm (+27.59%) and 1.22 ± 1.87 mm (+30.48%), respectively. The bone density was increased by a factor of 4, reaching an average of 387.47 ± 328.86 HU. Histomorphometric evaluations revealed new bone formation of 41.44 ± 5.37%, residual biomaterial of 24.91 ± 7.31%, and connective tissue of 33.64 ± 4.81%. The mean healing period was 8.32 ± 3.00 months.

**Conclusions** Data from this study confirmed the suitability of the tested materials in dental surgery. The biomaterials may be recommended for various clinical procedures. A satisfactory level of increase of new bone was reported in augmented sides. No significant differences were observed between the tested membranes. PLGA membranes might be superior to collagen membranes for their easier handling.

## Introduction

The biggest challenge facing treatments in implantology and maxillofacial surgery is achieving an adequate bone volume as well as a successful osteointegration for long-lasting results. Dental bone defects may be caused by trauma, genetic anomalies, cancer, age-related diseases, and in most cases as a natural physiologic process after tooth loss or extractions. 1 Thus, partial or complete edentulism leads in the corresponding region to bone loss and soft tissue changes which in turn mean unfavorable conditions for future prosthetic rehabilitation. Accordingly, the reconstruction of these alveolar ridge defects is indispensable by means of bone augmentation techniques, commonly assisted by bone substitute materials. Conventional bone grafts of biological origin are autografts (autogenous bone), allografts (homogenous bone), and xenografts (heterogeneous bone). All of them have their characteristics and advantages, but also limitations. 2 3 4 5 Alloplastic biomaterials are the synthetic nonosseous alternative, available in a biologically or nonbiologically derived form. 3 4 5 6 The most common and commercially offered alloplasts are calcium phosphate (CaP)-based graft substitutes or bioactive glass ceramics. 2 6 7 CaP materials are represented by hydroxyapatite (HA), α- and β-tricalcium phosphate (α-TCP and β-TCP), and biphasic calcium phosphate (BCP), a mixture of HA and β-TCP. The wide range of applications of alloplasts underlines their importance in the dental field of today: for coatings to stabilize implants, filling defects in oral and maxilla reconstructions, augmentations, and classical reconstructions of periodontal, periapical, or endodontic surgery. 6 7 8 9 10 11 Synthetic bone grafts have many beneficial qualities, including no risks of disease transmission, unlimited supply, biocompatibility, lower morbidity, and predictability. 1 7 9 The biological performance of alloplastic biomaterials depends on many factors such as chemical composition, morphology (for example, granule size, porosity, crystallinity), or mechanical stability. 2 3 9 These properties can be modified by thermal (sintering methods) or chemical treatments, 6 9 12 thus, bone regeneration can be directly influenced. 9 13 Generally, synthetic materials are described as having an osteoconductive behavior 3 4 7 with an osteoinductive potential. 14 Bone graft substitutes should resemble, in terms of macro- and microporosity structures, the natural human bone. 12 Porosity allows carrying out biological and biochemical processes like bone cell ingrowth, protein adsorption, and vascularization, and accordingly, interactions of the biomaterial surfaces with the surrounding tissue. 4 15 16 In guided bone regeneration (GBR), bone graft substitutes are combined with barrier membranes to maintain the stability of the augmented bone volume. Ideally, membranes should guide the slower migrating bone cells to the defect side, while preventing epithelial tissue ingrowth into the graft side. 1 In general, membranes can be classified as resorbable collagen membranes of porcine or bovine origin, synthetic resorbable poly(lactic-co-glycolic) acid (PLGA), and polylactide or nonresorbable expanded polytetrafluoroethylene membranes. Nowadays, numerous patients favor nonautogenous, synthetic bone graft substitutes due to ethical, cultural, or religious reasons. 17

The present study was focussed on the synthetic bone substitute granules (TIXXU GRAFT; Bredent Medical GmbH & Co. KG, Senden, Germany) and the synthetic bone putty (TIXXU GRAFT, Bredent Medical GmbH & Co. KG, Senden, Germany), coverd by either a synthetic PLGA membrane (TIXXU CONTROL, Bredent Medical GmbH & Co. KG, Senden, Germany) or a porcine collagen membrane (EZ Cure, Biomatlante, Vigneux-de-Bretagne, France). The hypothesis was that the tested materials have similar results to those described in the current literature suitable for the clinical procedures proposed.

## Materials and Methods

### Ethical Research in Humans

All patients involved had provided their informed consent prior to inclusion in the study. The experiments were performed under the guideline established by the Declaration of Helsinki as revised in 2008 for medical research involving humans. Possible side effects or complications would be immediately treated in accordance with current medical university knowledge. The study and all associated documents were approved by the Ethical Committee of the Catholic University of Murcia (UCAM).

### Study Design

Patients were recruited at the Department of Master's Degree in Implant Dentistry at the Dental Clinic of UCAM, and were treated from March 2018 to December 2020 within this project. The present study was an analysis of the records of 20 systemically healthy patients (7 females and 13 males), partially or completely edentulous, and finally restored with implants for prosthetic rehabilitation. The mean age of the patients was 54.09 ± 11.26 years (ranging from 38 to 75) at the time of surgery. The follow-up period was set as a minimum of a half year. In total, 35 clinical acts have been implemented, and 51 samples of the study material were used.

### This Study Included the Following Tested Materials

- TIXXU GRAFT (Synthetic Bone Substitute Granules, produced by Biomatlante, Vigneux-de-Bretagne, France, REF: TX0302G01, *n* = 23).
- TIXXU GRAFT (Injectable Synthetic Bone Substitute Putty, produced by Biomatlante, Vigneux-de-Bretagne, France, REF: TX1002PU50DE, *n* = 11).
- TIXXU CONTROL (Synthetic PLGA Membrane, produced by Biomedical Tissues, SAS, Nantes, France, REF: TICO2030, 20 × 30 mm, *n* = 10).
- EZ Cure (Porcine Collagen Membrane, produced by Biomatlante, Vigneux-de-Bretagne, France, 20 × 30 mm, REF: 0702EZC2030, *n* = 7).

All biomaterials were resorbable. They were obtained directly from the manufacturer in sealed packaging, and used without further treatments. The bone substitute grafts were produced with the MBCP technology with bioactive CaP (TIXXU GRAFT, Bredent Medical GmbH & Co. KG, Germany), categorized as biphasic material (BCP) with ratios of 60% HA [Ca _10_ (PO _4_ ) _6_ (OH) _2_ ] and 40% β-TCP [Ca _3_ (PO _4_ ) _2_ ] in the granule form. The putty version contained additionally a hydrogel (hydroxypropylmethylcellulose). BCP was obtained by sintering precipitation. 4 16 Data from in vitro studies 18 showed that the main elements of this material were calcium and phosphate in a ratio of approximately 1.5 to 2.2. The material had a total and intraparticle porosity of 74 and 32%, respectively. The pore size and distribution were around 65 to 72%, and the particle density was approximately 3.2%. The biomaterial had a micro- (<10 µm) and macroporous (100–600 µm) structure with documented grain sizes between 0.5–1 and 0.8–1.5 mm in diameter. The resorption rate was approximately 6 months. The following material was the fully synthetic membrane (TIXXU CONROL, Bredent Medical GmbH & Co. KG, Germany), made of PLGA. The tested membrane had a double-layered structure, fabricated in two processes of freezing and lyophilization with a PLGA solution. 19 The outer side of the membrane was covered by a smooth fascia of a dense glossy layer to prevent the ingrowth of gingival fibroblast cells and connective tissue (CT) invasion. The inner layer of the membrane had a porous matt fascia structure with nonwoven microfibers for the promotion of osteogenic cells and a controlled bone regeneration. The bilayer transmembrane structure allowed angiogenesis. According to the manufacturer's specifications, the resorption rate was around 6 months. The second membrane (EZ Cure, Biomatlante, France) had a cross-linked structure, originally from porcine. It was a commonly recommended collagen membrane for GBR with a documented resorption rate of around 3 months. 1 The used size of both membranes was 20 mm × 30 mm.

### Preoperative Examinations

Potential patients were informed about study conditions. All patients received careful periodontal examinations, including the assessment of supra- and subgingival plaque, gingivitis, probing depth, followed by oral hygiene instructions and, if indicated, periodontal therapy. The clinical cases were documented preoperatively, during the surgery, and postoperatively with full-HD pictures (camera: Canon EOS 200D, Japan; macro ring flash lite system: Meike MK-14EXT; macro lens 105 mm: Sigma F2.8 EX DG OS HSM, Japan).

### Radiological Analysis

Standardized three-dimensional radiographs were obtained by means of of a paralleling cone-beam computed tomography (CBCT) device (Orthophos SL 3D, Dentsply Sirona, United States/Germany), using a digital imaging software system (SIDEXIS 4, Dentsply Sirona). CBCT scans were taken before and at a minimum of 6 months after the surgery. For comparing the bone volume changes, parameters such as the length, width, and density of the recipient graft side were analyzed, first without, and finally with the applied material by using a dental planning software (Blue Sky Plan 4, United States). The alveolar bone of maxilla and mandible was measured in their entire length (basal–occlusal distance) and width (bucco-palatal/lingual distance) at three points. Density was measured in four random points. Always the same pre- and postoperative cross-sectional views were used for analysis in CBCT scans.

### Surgical Protocol

All surgeries were performed under local anesthesia, according to the manufacturer's protocol, in the maxilla ( *n* = 16) and mandible ( *n* = 5). Depending on the locus of the bony defect, five different clinical treatments were performed: sinus floor elevation ( *n* = 6), bone regeneration in the third molar region ( *n* = 1), socket prevention ( *n* = 15), ridge augmentation ( *n* = 11), and crest splitting ( *n* = 2). The materials were applied in the bone defects to augment and increase the bone volume; cases in combination with membranes, *n* = 14, and without, *n* = 6. The needed quantity of the materials was previously calculated in a patient's protocol, including the adjusted diameter, length, and amounts of the implants (Bioner Implant Systems, Barcelona, Spain), and a further prosthetic rehabilitation plan. In all clinical situations, implants were immediately inserted at the bone crest level, expected during the sinus lift augmentation. After the elevation of the sinus membrane, the implants were placed in the healed bone at least in 6 months' time. In total, 57 implants were placed during the study period in either the augmented sides or areas which were in close contact with biomaterials in the same jaw. After the surgeries, each patient received an anti-inflammatory treatment: 400 mg of ibuprofen every 8 hours for 3 days, and 0.12% chlorhexidine gel every 12 hours for 2 days. Patients were asked to follow the general guidelines after surgical procedures. Temporary prosthetic restoration was done. Sutures were removed after 8 to 10 days postoperative.

### Histologic Processing

Bone biopsies were taken with trephine needles after 6 months from the placement of biomaterials, processed for ground sectioning. In total, 11 biopsies were harvested. The protocol started with the fixation process. Samples were dehydrated in increasing grades of ethanol from 70 up to 100%, embedded with methacrylate, polymerized, and sectioned using a diamond saw (Exakt, Apparatebau, Norderstedt, Hamburg, Germany).

### Histomorphometry

The digital quantitative analysis was performed by using calibrated digital images, ranging from 4 *×* to 40 *×* magnification (Leica microscope Q500Mc, Leica DFC320s, 3,088 × 2,550 pixels, Leica Microsystems, Barcelona, Spain). The most central sagittal section of each implant was taken for histomorphometric analysis using MIP 4.5 software (Microms Image Processing Software, CID, Consulting Image Digital, Barcelona, Spain), and connected to a Sony DXC-151s 2/3-CCD RGB Color Video Camera. The areas of interest were marked, and their values were calculated digitally for the total percentage with the ImageJ software (W. Rasband, National Institutes of Health, Maryland, United States). The evaluation consisted of the measurements of new bone formation (NB), residual biomaterial (RB), and CT in relation to the total measurement area. Values were expressed in percentage.

### Statistical Analysis

Results were transferred into Excel (Microsoft Corp., Redmond, Washington, United States). Data analysis was performed with SPSS 20.0 software (New York, United States). The descriptive method was used to analyze the radiological mean differences in length, width, and density. Quantitative variables in the form of mean, median, standard deviation, maximum, and minimum were calculated for radiologic and histomorphometric evaluations. The paired *t* -test was deployed for the analysis of the differences within each group between baseline (preoperative) and final (postoperative) measurements. A *p* -value of <0.05 was established to be statistically significant.

## Results

The values followed a normal pattern of dispersion with a 95 percent confidence interval. Table 1 summarizes the statistically significant differences ( *p* < 0.05) between the measurements of the parameters pre- and postoperative. The univariate analysis showed a bone gain in length, width, and density of 27.59%, 30.48%, and 315.66%, respectively.

**Table 1:** Radiographic measurements of total alveolar bone in cross-sectional view in CBCT and evaluation

	Preoperative, mean ± SD	Postoperative, mean ± SD	*p* -Value
Length	15.59 ± 7.39 mm	16.55 ± 6.15 mm	0.00396 a
Width	9.67 ± 2.87 mm	10.89 ± 2.58 mm	1.112e ^−09^ a
Density	381.89 ± 384.47 HU	769.35 ± 378.40 HU	<2.2e ^−16^ a

Abbreviations: CBCT, cone-beam computed tomography; SD, standard deviation.

a Statistical significance: *p* < 0.05.

### Operation Day

In 76% of the cases, biomaterials were applied in the maxilla, and in 24% in the mandible, whereby their distribution was incidental. The planned prosthetic rehabilitations after substitute grafting and implant insertion were either a fixed hybrid acrylic prosthesis or crowns and/or bridges. All crowns and bridges were of metal-ceramic reconstruction.

### Postoperative Situation

**Fig. 1:**
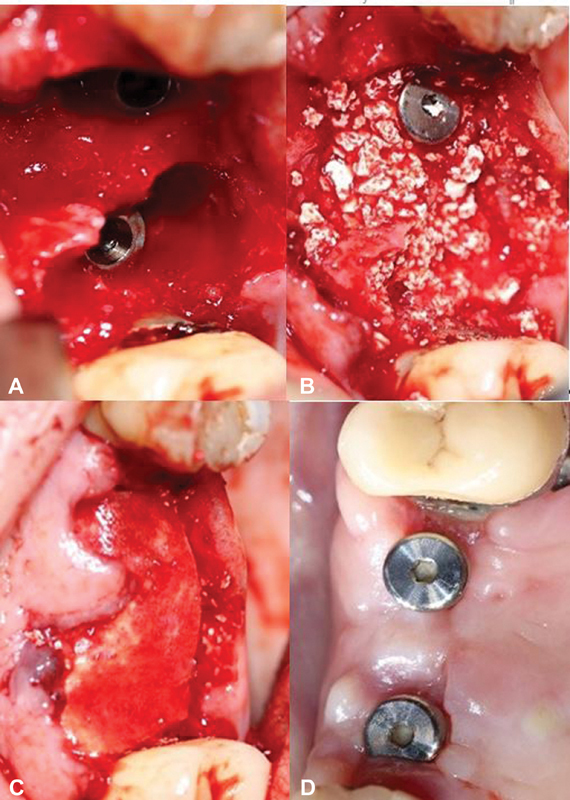
( **A** ) Extractions of teeth 15 and 16 and immediate implant insertions in the upper law; ( **B** ) applied bone graft granules; ( **C** ) covered by PLGA membrane; and ( **D** ) result after 6 months of healing time. PLGA, poly(lactic-co-glycolic) acid.

Seven of the 57 implants were lost in nonaugmented areas. In all other cases, uneventful healing was observed. Neither regional bony infections nor rejection of the biomaterials could be identified in clinical and radiographic examinations. Regarding the membranes from a clinical point of view, PLGA membranes were rigid under dry conditions, thus, easier to cut into the appropriate shape to cover the bony defects. After moistening, the mechanical flexibility increased, and a simple placement was possible. In comparison, the collagen membrane was softer, and stuck slightly at the instruments. The permeable cross-linked collagen structure was beneficial in allowing cell attachment from all sides, Attention should be paid to place the porous side of the PLGA membrane correctly on the bone surface without confusions of the sides. While suturing with soft tissue, the mechanical strength of both membranes seemed to be enough. The tested membranes did not disturb tissue healing or bone healing as demonstrated in Fig. 1 .

### Radiographic Evaluation

No signs of osteolysis were observed. The biomaterials were well included in the host bone, marked by increased radiodensity around it. The mean bone gain in length, width, and density was 0.96 ± 3.33 mm, 1.22 ± 1.87 mm, and 387.47 ± 328.86 HU, respectively ( Table 2 ).

**Table 2:** Mean bone gain in length, width, and density postoperative

Title 1	Length [mm]	Width [mm]	Density [HU]
*n* a	315	315	420
Range	14.81	10.12	1,524.75
Mean	0.96	1.22	387.47
SD	3.33	1.87	328.86
Minimum	−4.71	−1.44	−281
Maximum	10.10	8.68	1,243.75
Median	0.17	0.74	403.75

a Number of measurements.

### Morphometric Evaluation

**Fig. 2:**
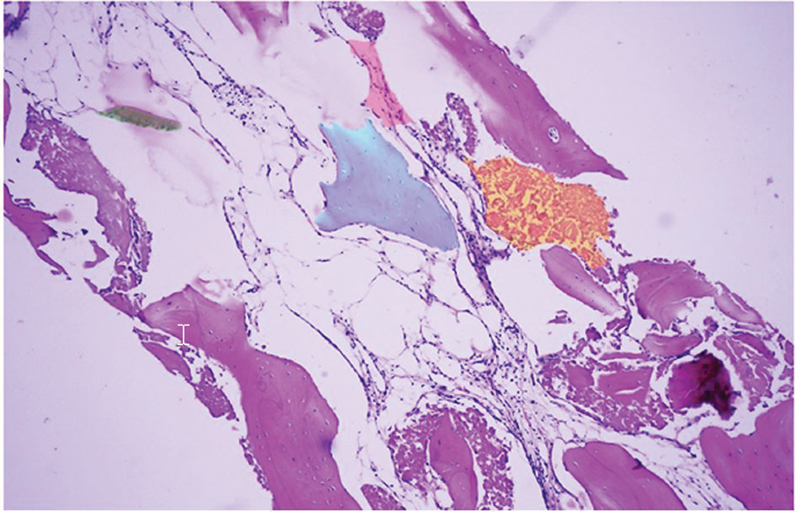
Example of the macroscopic image (magnification 10 *×* *)* . Blue: residual biomaterial; orange: absorption; rose: connective tissue; green: new bone formation.

New bone regeneration and osteoclastic activity, detected by the absorbed lacunae of the inserted biomaterial, were observed ( Fig. 2 ). In all samples, the newly formed bone was in close contact with the graft material. The biomaterial was surrounded by CT. Percentage values of NB, RB, and CT are listed in Table 3 .

**Table 3:** Percentages of NB, RB, and CT after a healing period of 6 months

%	Mean	Median	SD
New bone formation	41.44	42.61	5.37
Residual biomaterial	24.91	27.37	7.31
Connective tissue	33.64	33.52	4.81
Total	99.99		

Abbreviations: CT, connective tissue; NB, new bone formation; RB, residual biomaterial; SD, standard deviation.

## Discussion

One of the first extensive studies by Daculsi et al 20 demonstrated the efficiency of BCP as a grafting material of MBCP technology covering bone defects approximately 30 years ago. Generally, the biodegradation mechanism of synthetic bone substitute grafts is introduced by osteoclast-induced resorption of the material, resulting in a release of calcium and phosphate, and an increase of these elements in the newly formed bone that is close to the substitute. 6 9 Osteoclasts have the same behavior at the implantation side as at the bone surface. High extracellular calcium levels stimulate osteogenesis in tissue by attracting osteoblasts. A successful osteointegration includes the replacement by autologous tissue after biomaterial degradation. 6 *In vivo* studies by Yamada et al 21 confirmed that BCP (60% HA/40% β-TCP) might be seen as the satisfactory mixture for the clinical application. HA operated for a longer time as a mechanical strong scaffold regarding its slow 1 to 2 years resorption rate, 1 4 21 whereas β-TCP was approximately 10 to 20 times faster resorbed due to its higher calcium concentration during the first 3 months. 1 22 The properties of the combination of both materials were comparable to the biological degradation rate of natural human bone. 23 Moreover, comparing the histomorphometric results of the present study with data from the literature while respecting similar healing periods, Friedmann et al 24 obtained 38.8% NB in ridge augmentations and maxillary sinus grafting. Mangano et al 25 used BCP produced by the same MBCP technology in a clinical study for sinus augmentation and obtained 28.3% NB. The systematic review and meta-analysis of Danesh-Sani et al 26 compared histomorphometric results after sinus floor augmentation of all types of grafting materials in different healing times from 136 studies. Regarding the healing period of 4.5 to 9 months, the autogenous bone had the highest amount of NB (43.15%) compared with other graft materials, followed by alloplastic materials (29.27% NB), and allografts with the lowest amount of NB (25.02%). Notably, alloplastic materials were not clearly differentiated. In the randomized controlled trial by Schmitt et al, 27 four different grafting materials were compared in 30 patients after 5 months in sinus augmentations: Straumann Bone Ceramic (BCP), Bio-Oss (anorganic bovine bone), Puros (mineralized cancellous bone allograft MCBA), and autologous bone. The autograft reported the highest amount of NB (42.74%), followed by MCBA with 35.41% and BCP 30.28%. The bovine bone revealed the lowest amount of NB (24.90%). Further studies compared the efficacy of BCP against autogenous bone and concluded higher results in NB with autografts: 38.63 to 41% 28 or 36.8% 29 versus NB in BCP of 26.68 to 33.70% 28 or 28.2%. 29 Taking all results into consideration, it is remarkable that the present study demonstrated the capacity of the novel biomaterials to promote higher amounts of NB compared with conventional BCPs, xenografts, and allografts. 24 25 26 27 The tested BCP reached similar high amounts of NB to autografts and demonstrated the resemblance in behavior to autogenous bone. 27 28 29 One disadvantage of autogenous bone is the higher rate of bone formation initially, but in turn also a faster resorption rate. 30 In contrast to the rougher surface of autologous bone, BCP has initially a smooth surface which has to be prepared by macrophages for improving the bone cell apposition. 30 Lee et al 22 observed the first bone formation at around 12 weeks after implantation. BCP inhibited a too early osteoclastic resorption 22 ; therefore, BCP seems to be more stable and predictable in behavior. In a systematic review, Troeltzsch et al 31 stated the clinical efficacy of grafting materials in alveolar ridge augmentations. After an investigation of a total of 184 papers, a horizontal mean bone gain of 2.2 ± 1.2 mm was described for synthetic biomaterials. The result is comparable to the mean width of 1.22 ± 1.87 mm of the present study. The highest vertical gain of 4.5 ± 1 mm was reached by autogenous bone mixed with allogenic or xenogeneic grafts, and the overall mean of all grafts was 3.7 ± 1.2 mm. 31 Accordingly, based on the present study, one might conclude that augmentation with alloplasts, in the form of granules and/or putty, is recommendable with a confirmed satisfactory outcome up to a height of 3 to 4 mm. For augmentations with synthetic particulate bone graft higher than 4 mm, further clinical studies are necessary. Regarding the membranes, some studies underlined better outcomes for GBR using barrier membranes, 32 and others showed no significant differences. 33 In the present study, the results of radiographic and histomorphometric evaluations according to the PLGA and collagen membrane did not differ substantially, and both membranes had good outcomes as physical and biological barriers. In general, no systemic toxicity has been observed yet for PLGA membranes, 34 and collagen membranes are cytocompatible because of their natural origin. 35 Hoornaert et al 36 figured out that without using additonal bone grafting materials, PLGA membranes have a higher bone regeneration rate of 30% NB, compared with cross-linked porcine collagen membranes (24.6%). PLGA was hydrolyzed after 6.5 months, whereby collagen membranes were already completely resorbed in around 8 weeks. Collagen membranes have faster degradation rates depending on different factors, including the tissue of origin or mechanical properties. 36 Alveolar bone healing was marked at around 4 months 36 ; therefore barrier membranes should sustain its biological function during this period. Consequently, PLGA membranes might be safer, and have a better predictable resorption rate. The *in vitro* and *in vivo* studies of Won et al 37 showed a similar level of biocompatibility and bone regeneration potential of PLGA and collagen membranes (NB: 24.26 and 13.84%, respectively). The study asserted that PLGA membranes were more reliable in retaining form in the oral cavity than collagen membranes, which lost a tad of stability under wet conditions. Finally, the percentages of rate of rehabilitation of dehiscence defects for PLGA and collagen membranes were reported in the literature to be 70.20 and over 80%, respectively, whereby the complication rates were the highest for PLGA (37.4%) and lowest for the collagen membrane (10.4%). 31 Summing up all data from the present and previous studies, 31 32 33 34 35 36 37 apart from small differences, both types of membranes are biocompatible and suitable for GBR. Overall, BCPs have a great potential in dental treatments. To optimize the regenerative capabilities of bone, some studies led to promising results by adding bioactive agents, including silver nanoparticles, 38 advanced platelet-rich fibrin, 39 or mesenchymal stem cells. 40 In future perspectives, more attention should be focused to hone the skills of synthetic BCPs in combination with tissue engineering and cell culture technology.

## Conclusions

With the limitations of a clinical study in humans, the tested novel synthetic materials can be recommended for different dental surgical treatments, and may be an adequate alternative to autogenous bone. The present study reported an increased level of newly formed bone compared with current data from the literature. A statistically significant bone gain in length, width, and density in augmented sides could be observed after 8 months. It has been proven that both tested membranes have fulfilled all biological and mechanical functions for daily work. Further studies are necessary to verify the clinical effects of these biomaterials for long-term results as well as for patients with medical history or medical risks.
